# Sequence statistics of tertiary structural motifs reflect protein stability

**DOI:** 10.1371/journal.pone.0178272

**Published:** 2017-05-26

**Authors:** Fan Zheng, Gevorg Grigoryan

**Affiliations:** 1 Department of Biological Sciences, Dartmouth College, Hanover, NH, United States of America; 2 Department of Computer Science, Dartmouth College, Hanover, NH, United States of America; 3 Department of Chemistry, Dartmouth College, Hanover, NH, United States of America; Indian Institute of Science, INDIA

## Abstract

The Protein Data Bank (PDB) has been a key resource for learning general rules of sequence-structure relationships in proteins. Quantitative insights have been gained by defining geometric descriptors of structure (e.g., distances, dihedral angles, solvent exposure, etc.) and observing their distributions and sequence preferences. Here we argue that as the PDB continues to grow, it may become unnecessary to reduce structure into a set of elementary descriptors. Instead, it could be possible to deduce quantitative sequence-structure relationships in the context of precisely-defined complex structural motifs by mining the PDB for closely matching backbone geometries. To validate this idea, we turned to the the task of predicting changes in protein stability upon amino-acid substitution—a difficult problem of broad significance. We defined non-contiguous tertiary motifs (TERMs) around a protein site of interest and extracted sequence preferences from ensembles of closely-matching substructures in the PDB to predict mutational stability changes at the site, ΔΔ*G*_*m*_. We demonstrate that these ensemble statistics predict ΔΔ*G*_*m*_ on par with state-of-the-art statistical and machine-learning methods on large thermodynamic datasets, and outperform these, along with a leading structure-based modeling approach, when tested in the context of unbiased diverse mutations. Further, we show that the performance of the TERM-based method is directly related to the amount of available relevant structural data, automatically improving with the growing PDB. This enables a means of estimating prediction accuracy. Our results clearly demonstrate that: 1) statistics of non-contiguous structural motifs in the PDB encode fundamental sequence-structure relationships related to protein thermodynamic stability, and 2) the PDB is now large enough that such statistics are already useful in practice, with their accuracy expected to continue increasing as the database grows. These observations suggest new ways of using structural data towards addressing problems of computational structural biology.

## Introduction

Quantifying sequence-structure relationships in proteins has been a long-standing fundamental challenge in computational structural biology. In principle, a molecular mechanics (MM) description of the protein molecule offers the potential to estimate arbitrary thermodynamic parameters via statistical mechanics, as a function of sequence, but this suffers from issues of computational tractability [1–3]. Thus, a variety of empirical scoring functions have been developed for representing sequence-structure compatibility, offering reduced computational complexity at the expense of physical correctness [4–7]. Among these, knowledge-based potentials (also known as statistical potentials) have derived quantitative insights from statistics of native structures in the Protein Data Bank (PDB). The power of these approaches lies in their ability to treat relaxations of protein degrees of freedom implicitly, by drawing on structural observations in the PDB. For example, rotameric probabilities or backbone *ϕ*/*ψ* angle frequencies represent entire ensembles of similar conformations rather frozen structures [8]. A similar argument applies to distance bins in atomic or residue-level interaction potentials [9]. Knowledge-based potentials have contributed significantly towards progress in such grand challenges as structure prediction, and a broad variety of structural features have been exploited towards attaining better statistical potentials—e.g., backbone dihedral angles, atomic distances and densities, bond orientations, residue burial states, and inter-residue contacts, to name a few [9–14]. Statistics of these structural features, and their associated sequence propensities, have been combined into scoring functions in a variety of ways [9, 15–19].

Simple local structural descriptors, such as distances or angles, have the advantage that their individual statistics are well estimated from available structural data. On the other hand, combining the statistics of such descriptors into a single scoring function effectively amounts to estimating (either directly or indirectly) their joint probability distribution, which is not trivial due to inter-dependencies between descriptors. It is often necessary to imprecisely assume independence among features to produce generalized models. This limitation can be potentially addressed by using larger structural motifs in place of simple descriptors, such that the former would inherently capture correlations between elementary features. Further, a motif-based description of sequence-structure relationships would recognize the modular nature of protein structural patterns, the evidence for which across a range of scales has been mounting in recent studies [20–28]. Sequence-structure relationships extracted from modular contiguous backbone fragments have been instrumental in the advancement of structure prediction and protein design—e.g., as part of Rosetta [29–31] and I-TASSER [32, 33] methods. In recent years, modularity in structural motifs that extend beyond secondary structure has also been described and suggested to be of use in structural modeling and analysis [20–22, 34–36]. In a notable example, Feng and Barth have systematically analyzed structural motifs in multipass membrane proteins, having found that units of three interacting transmembrane helices mostly fall into six structural classes [21]. The authors went on to shown that the sequence-structure relationships implied by these motifs are predictive of topology and conformational flexibility [21]. Our own prior work has found that the protein structural universe is highly degenerate at the level of multi-segment tertiary motifs (TERMs), with emergent sequence-structure relationships able to predict native-like sequences given previously unseen native backbones [20].

At the extreme, motifs can be defined to represent the entire relevant structural environment, capturing the exact context, but statistics may become limiting. Thus, there is a tradeoff between accuracy and interpretability of statistics as motifs become more complex and detailed. So where is the optimum? In this work, we argue that at the current size of the structural database, it is already practical to quantify sequence-structure relationships using multi-segment TERMs, by extracting sequence models from ensembles of closely matching substructures. To this end, we focus on the task of predicting changes in protein stability upon point mutations, ΔΔ*G*_*m*_. We show that quantitative estimates can be derived using information from TERMs alone, with resulting accuracy on par with or better than that of statistical potentials and state-of-the-art estimators.

To date, best performance in ΔΔ*G*_*m*_ prediction has been reported for machine-learning methods that combine multiple features and train on large sets of experimentally measured ΔΔ*G*_*m*_ values [37–42]. Despite notable successes, assessments of existing methods have concluded that none consistently make accurate predictions on diverse test sets, showing there to be significant room for improvement and motivating the development alternative approaches [43, 44]. Here we show that sequence statistics of TERMs surrounding the mutated position, emergent from closely matching substructures in the PDB, enable ΔΔ*G*_*m*_ prediction on par with state-of-the-art methods, while requiring no training on available data. Further, this estimator outperforms all tested competing models when applied on an unbiased dataset of diverse mutations from recent deep sequencing analyses [45]. In addition, we show that prediction accuracy is driven by the the amount of available structural data, enabling the method to estimate the confidence level of individual predictions. Taken together, our results show that tertiary structural motifs and their PDB-based sequence statistics encode sequence-structure relationships reflective of fundamental thermodynamics of structure. This suggests that data mining strategies with complex multi-segment motif should produce increasingly useful and accurate insights as the structural database continues to grow.

## Theory

**Fig 1:**
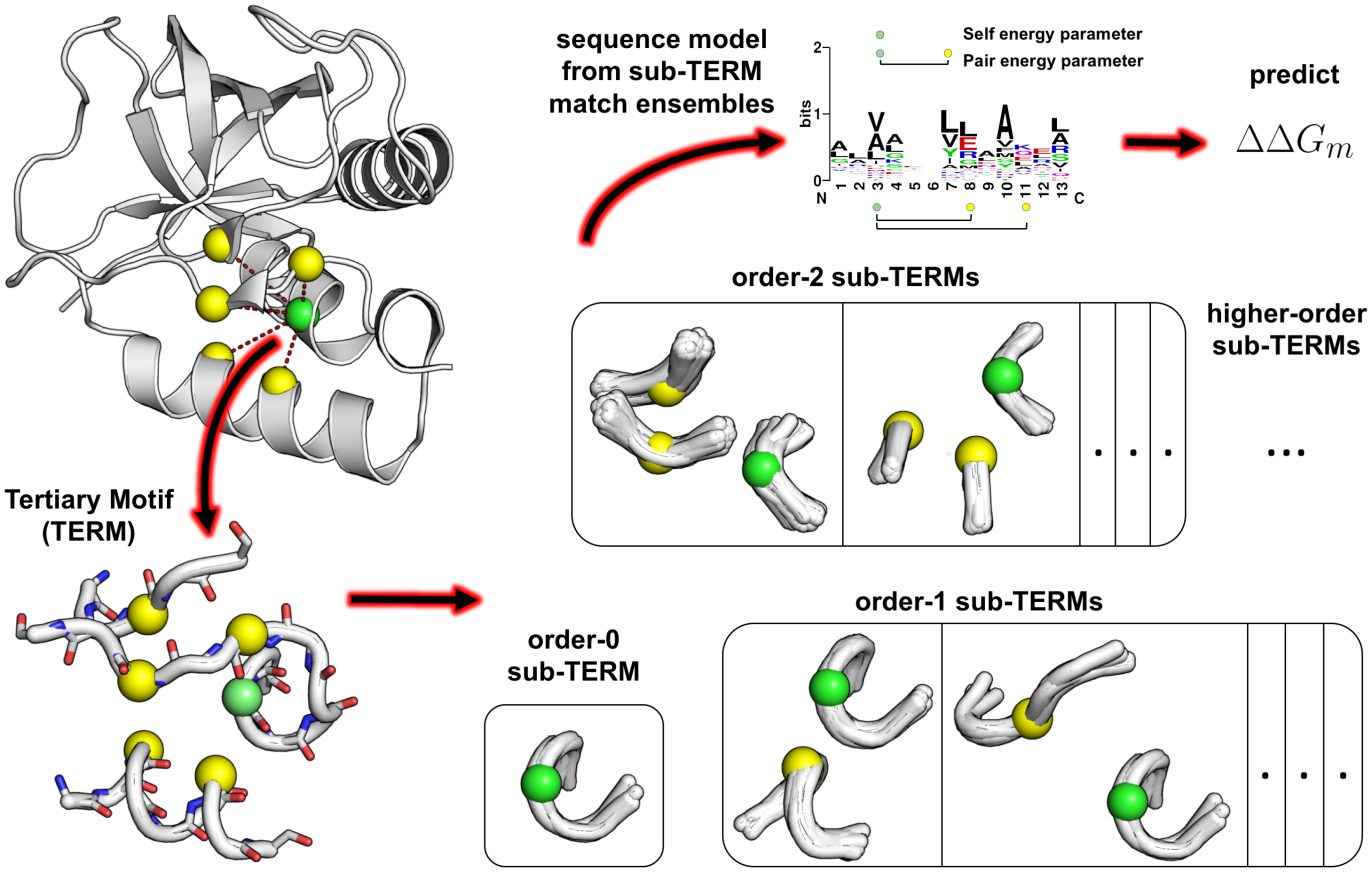
TERM-based ΔΔ*G*_*m*_ prediction. Procedural flow is indicated with arrows, starting from the top left. Given a structure of the protein of interest, a TERM is defiend around the mutated position (green sphere) to include any potentially contacting positions (yellow spheres) and flanking backbone segments (white sticks and ribbon). The TERM is next decomposed into sub-TERMs—i.e., substructures containing a subset of the contacting positions and flanking segments. Structural ensembles for each sub-TERMs are generated by searching the PDB for close structural matches using MASTER [47]. Finally, sequences from matching ensemble of all sub-TERMs (and the original TERM, data permitting) are used to extract positional and pair amino-acid preferences to predict ΔΔ*G*_*m*_.

In previous work, we proposed the local tertiary motif (TERM) as a compact elementary unit of protein structure [20, 46]. Briefly, a TERM is defined around a “seed” position *i* to include its surrounding backbone segment and those of all positions poised to contact *i*—i.e., positions capable of hosting side-chains that can contact side-chains at *i* (Fig 1A; see Materials and methods). Thus, a TERM is (generally) a multi-segment motif representing the local structural environment surrounding its seed residue. Close structural matches to a given TERM, from unrelated proteins, can be seen as reporting on the sequence determinants of the TERM’s backbone geometry, under a certainly amount of implicit structural relaxation. Here we test wether these TERM statistics reflect fundamental determinants of structural stability by using them to predict ΔΔ*G*_*m*_ of point mutations.

Suppose we seek to estimate the change in stability upon mutating the protein position *i* from amino acid *u* to *v*, $\Delta \Delta G_{u,v}^{i}$. If the TERM seeded at residue *i* has many diverse structural matches in the PDB, we may be able to deduce from the sequences of these matches whether *u* or *v* is energetically preferred at *i*, and by how much. In the limit of a large number of matches, it would be possible to derive a full statistical model for the TERM, describing the probability of observing it with any given amino-acid sequence. Then, the relative stability of *u* versus *v*, in the specific context of the protein in question, could be directly evaluated. Of course, such a complete model could not be obtained in many realistic cases, where the number of matches may be limited. But approximations involving a subset of positions in the TERM may be plausible. In the limit of very few available matches, the mere overall frequency of *u* versus *v* at *i* may still be informative. The approach we take here aims to build as complete and accurate of a model for the sequence preferences at and around *i* as possible, based on the totality of available structural match data (Fig 1).

### General formulation

Here we assume that the only structural and sequence context that influences $\Delta \Delta G_{u,v}^{i}$ is captured by the TERM seeded at residue *i*; lets call it *t*. This includes the backbone ensemble represented by *t* and the amino acids at protein positions covered by *t* (excluding *i*), $\vec{\sigma }$. We thus express $\Delta \Delta G_{u,v}^{i}$ as:

$$\Delta \Delta G_{u,v}^{i}=E^{i}\left(v|\vec{\sigma },t\right)-E^{i}\left(u|\vec{\sigma },t\right)$$ (1)

where $E^{i}\left(x|\vec{\sigma },t\right)$ is the effective statistical energy of amino acid *x* in the local sequence and structural environment of position *i*. Further, we assume this statistical energy to be composed of only effective self and pair energy contributions—energy parameters (EPs):

$$E^{i}\left(x|\vec{\sigma },t\right)=J_{x}^{i}+\sum_{j\in C}J_{x,{\vec{\sigma }}^{j}}^{ij}$$ (2)

where $J_{x}^{i}$ is the effective self energy parameter (sEP) describing the compatibility of *x* at position *i* with the backbone ensemble of *t*, each $J_{x,{\vec{\sigma }}^{j}}^{ij}$ represents an effective pair energy parameter (pEP) describing the correction to $J_{x}^{i}$ associated with the sequence context at position *j* (i.e., the amino acid ${\vec{\sigma }}^{j}$), and *C* is the set of positions within *t* that are most likely to influence amino-acid preferences at *i*. Here, we take *C* to contain the positions capable of accommodating contacts with *i*, defined via our previously presented pseudo-distance metric of “contact degree” [46] (see Materials and methods).

Our goal is to deduce EP values from the ensembles of structural fragments whose backbone conformations closely resemble *t*. We do so by seeking EPs that maximally describe the sequences observed within these matches. Identifying matches is made simple with our structure search engine MASTER [47], which can find all sub-structures from a structural database that are within a given backbone root-mean-square-deviation (RMSD) cutoff of *t* (see Materials and methods and S1 Text). However, complex TERMs may not have sufficiently many examples in the PDB to support our goal of extracting meaningful EPs. For this reason, we also consider substructures of *t*, which we refer to as “sub-TERMs”, and use the combined statistics of multiple sub-TERMs of varying sizes and complexities to approximate the sequence-structure relationships of the original *t*.

### Motif definition and statistics extraction

An order-*k* sub-TERM of *t* is a substructure that includes residue *i*, with its local backbone segment, and *k* residues from *C* (0 ≤ *k* ≤ |*C*|), with their local backbone segments. Thus, a total of 2^|*C*|^ sub-TERMs are possible, each induced by a unique subset of contacting residues, and all of these can be searched within the PDB to generate matching ensembles. However, we reasoned that higher-order sub-TERMs are unlikely to be well represented in the PDB if lower-order sub-TERMs contained within them are not. Thus, to limit computational time while preserving most of the relevant data, we initially searched for the elementary order-0 TERM (i.e., the local backbone segment around *i*) and all order-1 sub-TERMs, with each order-*k* sub-TERM (*k* ≥ 2) subsequently searched only if the number of matches for all of its constituent lower-order sub-TERMs exceeded 100. Structural matches were identified with MASTER [47], using a backbone RMSD cutoff that varied based on the size and complexity of the query TERM [20] (see Supplementary Methods in S1 Text). Matches were then filtered to remove those originating from proteins homologous to the protein with the analyzed mutation, so as to remove any unfair bias towards the native residue. Remaining matches were clustered based on their local sequence similarity around the mutated position, with only one representative match from each cluster preserved as a redundancy filter (see Materials and methods). This final set of matches was used for extracting sequence statistics and building a model to predict ΔΔ*G*_*m*_, as detailed below.

### Deducing energy parameters from match sequence statistics

Let *m* be a specific structural match to the sub-TERM *t*_*s*_ induced by contacting positions *C*_*s*_ ⊆ *C*, and let ${\vec{\sigma }}_{m}$ be the sequence of that match. Then, the effective energy of amino acid *x* at the seed position *i* in the context of this match can be represented following Eq 2 as:

$$E_{m}^{i}\left(x|{\vec{\sigma }}_{m},t_{s}\right)=J_{x}^{i}+\sum_{j\in C_{s}}J_{x,{\vec{\sigma }}_{m}^{j}}^{ij}$$ (3)

where ${\vec{\sigma }}_{m}^{j}$ is the amino acid in the *j*-th position of match *m*. From this, we can compute the probability of observing *x* at *i* in match *m*, $P_{m}^{i}\left(x\right)$, under our statistical energy model as:

$$P_{m}^{i}\left(x\right)=\frac{1}{Z_{m}}\cdot \exp\left[-E_{m}^{i}\left(x|{\vec{\sigma }}_{m},t_{s}\right)\right]\cdot P_{0}\left(x\right)$$ (4)

where *P*_0_(*x*) represents the background bias frequency of observing amino acid *x* and *Z*_*m*_ is the is the partition function over all possible amino acids:

$$Z_{m}=\sum_{a=1}^{20}\exp\left[-E_{m}^{i}\left(a|{\vec{\sigma }}_{m},t_{s}\right)\right]\cdot P_{0}\left(a\right)$$ (5)

The background frequency *P*_0_ accounts for the fact that different amino acids may have different *a priori* proposal probabilities due to reasons unrelated to either structure or function (e.g., due to fundamental rates of genomic nucleotide conversions). Biases in amino-acid distributions that appear in even non-coding intergenic DNA regions suggest that such a background is likely non-uniform [48]. On the other hand, such biases vary by species, so an exact definitive background is difficult to estimate. Still, Echols *et al*. showed that intergenic and genomic amino-acid distributions, across species, do agree in their overall trends—e.g., over-representations of such amino acids as Leu and Ser, and under-representation of Trp, Cys, and Tyr [48]. Thus, in our study, we took as *P*_0_ the overall distribution of amino acids in the structural database (see Table 1 in S1 Text), such that contributions from different species, folds, and structural environments would average out to some extent.

Using Eq 4, we can compute the distribution of amino acids we expect at the seed position *i* in matches to any sub-TERM *t*_*s*_, given a set of EP values. We then propose that the optimal EP values are those that maximally describe the amino acids actually observed at the seed position in these matches. That is, those parameters for which probabilities emergent from Eq 4 are maximally coincident with observed frequencies. Thus, we express the problem of estimating optimal EP values as minimization of the following objective function:

$$L_{0}=-\frac{\sum_{s=1}^{S}w_{s}\frac{1}{M_{s}}\sum_{m=1}^{M_{s}}\ln P_{m}^{i}\left(\sigma_{m}^{i}\right)}{\sum_{s=1}^{S}w_{s}}$$ (6)

where *S* is the total number of sub-TERMs considered, *M*_*s*_ is the number of matches for the *s*-th sub-TERM, and *w*_*s*_ is a weighting factor of the *s*-th sub-TERM that reduces the contribution of sub-TERMs with fewer matches as these may have unreliable statistics. We set *w*_*s*_ to *M*_*s*_/(*M*_*s*_ + 1000), such that 1,000 matches was the characteristic cutoff around which sub-TERM contributions changed significantly.

### Robustness of prediction under sparse statistics

Our model needs to estimate 20 + 400 ⋅ *n* parameters for a position with *n* contacts, while not all of these may be equally well represented in sub-TERM matches. The objective function in Eq 6 alone may not guarantee robust estimation of all EPs. In an extreme case, when a certain amino acid is never observed in an ensemble, optimal values of associate EPs would be infinite, which is unrealistic. More generally, EP estimations are susceptible to stochastic noise if relevant observations are sparse. We address this by introducing prior EP values (i.e., starting assumptions) and a regularization term to the objective function that keeps EPs close to these priors, unless considerable evidence exists to suggest otherwise. The full objective function becomes:

$$L=L_{0}+\frac{\lambda }{\left|P\right|}\sum_{p\in P}^{\left|P\right|}\frac{{\left(J\left(p\right)-\hat{J}\left(p\right)\right)}^{2}}{O(p)+1}$$ (7)

where *L*_0_ is taken from Eq 6, λ is a parameter to control the strength of regularization, *P* is the set of all EPs, *J*(*p*) and $\hat{J}\left(p\right)$ are the current and prior values of a given parameter *p*, and *O*(*p*) is a measure of *occurrence* of *p*—i.e., the number of times the sequence pattern represented by *p* is observed in matches over all ensembles. For example, the occurrence of an sEP $J_{x}^{i}$ would be the number times *x* is observed at position *i* within all matches. We set the default values for all sEPs to be zeros, and those for pEPs to be equal to a generic residue-level contact potential derived from observations of amino-acid pairs at positions poised for interaction (see Materials and methods), unless otherwise specified. The impact of the modified objective in Eq 7 is that the estimation of EPs is driven by the data when relevant matches are abundant, but defaults to prior values smoothly when observations are too sparse.

## Results

### Predicting ΔΔ*G*_*m*_ of point mutations

To benchmark the performance of the above framework we used point mutations from the ProTherm database [49] with experimentally-measured ΔΔ*G*_*m*_ values and available structures in the PDB. Specifically, we used the subset of ProTherm curated by Dehouck *et al*. (mutations in globular proteins with values averaged over redundant entries), with a total of 2,648 point mutations at 1,428 unique protein sites from 115 proteins; hereafter referred to as S2648 [37]. This dataset covers mutations across diverse environments with respect to TERM-based statistics, as shown in Fig 1 in S1 Text (e.g., the number of contacts and sub-TERMs per position, and the number of matches per sub-TERM vary considerably). The set thus represents a robust test of whether TERMs capture relevant sequence-structure relationships.

**Fig 2:**
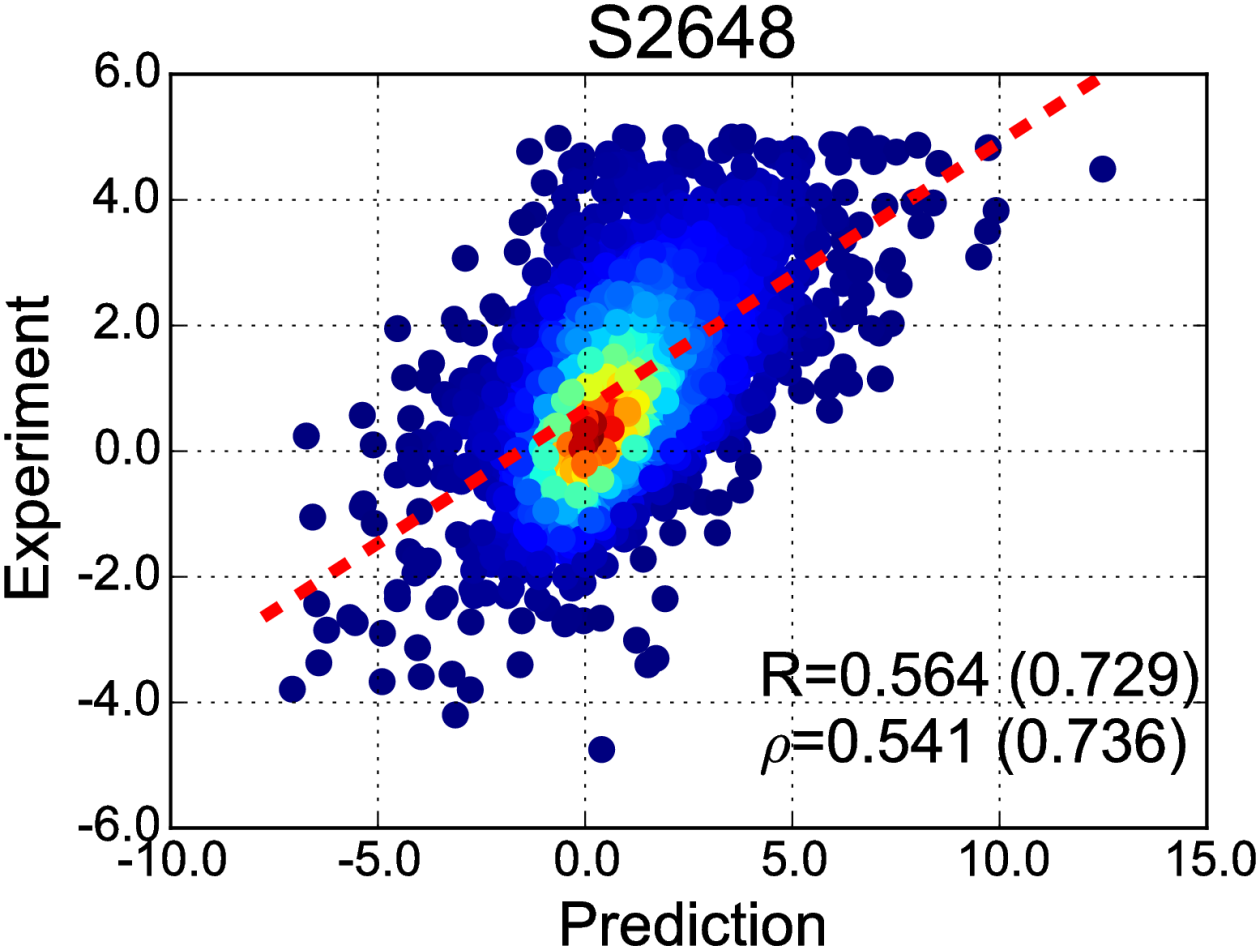
The performance of TERM-ΔΔ*G*_2_ on S2648. Predicted and measured ΔΔ*G*_*m*_ values are plotted on the X- and Y-axes, respectively. Color represents point cloud density. The least-squares regression line is shown with dashes.

**Fig 3:**
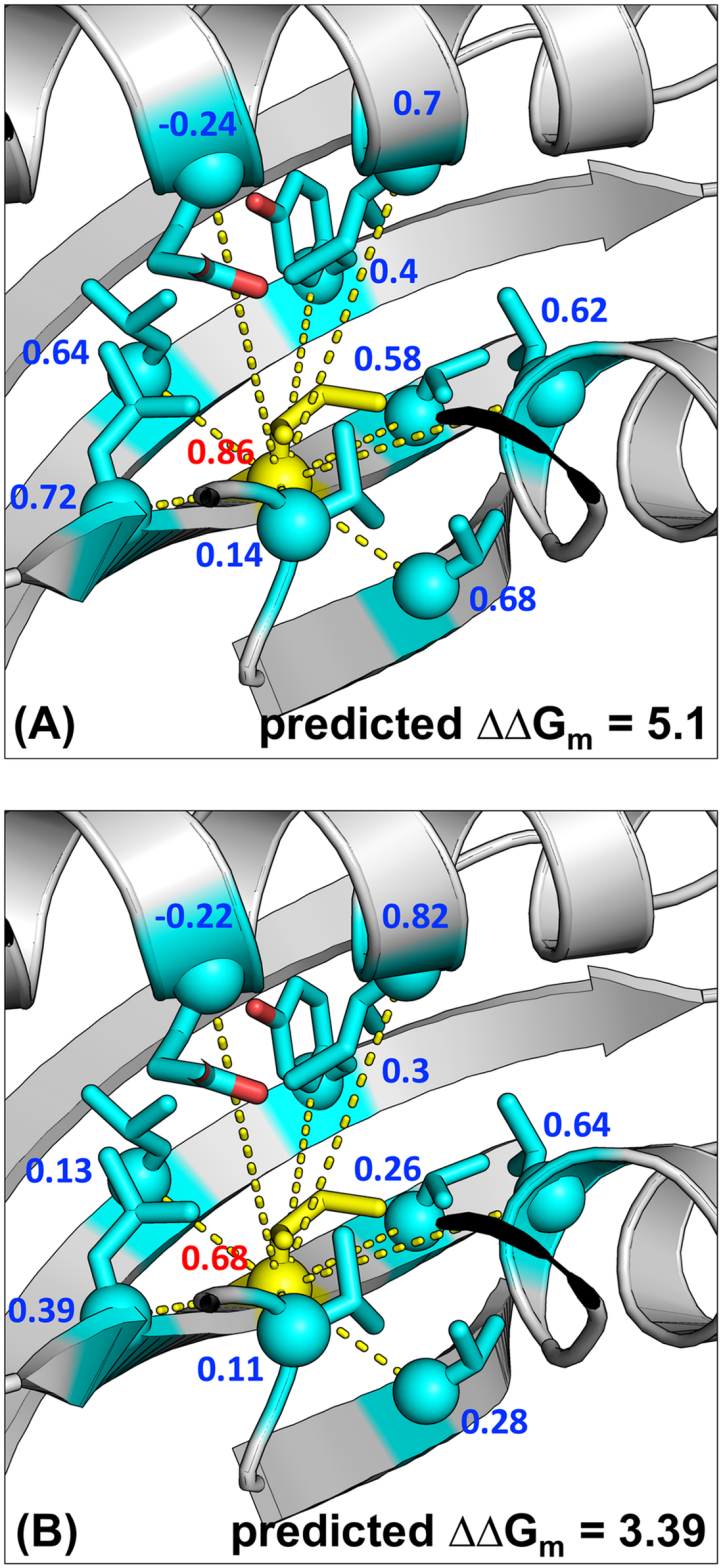
The role of multi-contact ensembles in ΔΔ*G*_*m*_ prediction, on the example of 1RIS_AI8A. (A) and (B) correspond to models TERM-ΔΔ*G*_1_ and TERM-ΔΔ*G*_2_, respectively. The mutated position is shown in yellow and all its contacting positions (9 in total) are shown in cyan. Values of estimated sEP and pEPs are shown in red and blue, respectively. The experimental ΔΔ*G*_*m*_ for the mutation is 3.56 kcal/mol (destabilizing).

The only free variable in our framework is the regularization parameter λ in Eq 7, which can be interpreted as the characteristic number of occurrences a given EP must reach before it can be allowed to significantly deviate from its default value. We thus reasoned that a value of λ = 1,000 maybe be roughly appropriate (e.g., for pair parameters, there are a total of 400 possible amino-acid combinations, so more matches should be needed to confidently resolve their relative effects). With this choice of λ we referred to our model as TERM-ΔΔ*G* and used it to predicted the ΔΔ*G*_*m*_ for all mutations in S2648; see Fig 2 in S1 Text. Throughout the paper, performance is assessed by comparing predicted and experimentally-measured ΔΔ*G*_*m*_ values via the Pearson correlation coefficient (*R*), the Spearman rank-order correlation coefficient (*ρ*), the root mean square of the residuals after linear regression (RMSE), and the fraction of correctly predicted ΔΔ*G* signs (Accuracy) (see Materials and methods). The performance on S2648 showed *R* = 0.56, *ρ* = 0.54, RMSE = 1.21 and Accuracy = 75%. To test how sensitive this result was to the choice of λ, we repeated all calculations with several other values, confirming that the initial intuitive choice was in the right range and that, in general, differences were small until λ deviated considerably from the 200-1,000 range (see Fig 3 in S1 Text and Table 1).

**Table 1:** Prediction performance with different strengths of regularization.

λ	*R* ^a^	*ρ* ^a^	RMSE	Accuracy
5	0.499 / 0.689	0.505 / 0.712	1.258	0.748
20	0.531 / 0.708	0.527 / 0.724	1.230	0.754
50	0.542 / 0.714	0.534 / 0.729	1.220	0.756
200	0.550 / 0.717	0.540 / 0.732	1.212	0.748
500	0.554 / 0.718	0.542 / 0.734	1.209	0.748
1000	0.556 / 0.720	0.541 / 0.734	1.207	0.745
5000	0.555 / 0.719	0.535 / 0.730	1.208	0.735
10000	0.551 / 0.716	0.528 / 0.723	1.211	0.735

^a^ The second number in columns *R* and *ρ* is the correlation coefficient after removing 10% of data points as outliers.

Still, the performance did decrease when λ was either very low or very high (Table 1)—i.e., when either EP priors or TERM statistics were given little weight, respectively, suggesting that both were important for good prediction accuracy. To explore this further, we formulated two control models: one ignored TERM statistics and simply set EPs to their default values, amounting to a simple residue-level statistical contact potential (model ConPot), and another assumed no prior knowledge on EPs, setting their default values to zero, and used TERM statistics to derive parameters as described above (model TERM-ΔΔ*G*_*x*_); see Table 2. Both models performed significantly worse than the initial model in all metrics. These results suggest that contact potential values and TERM statistics may complement each other—the former are much better defined statistically, but lack any structural context, whereas the latter have considerable contextual information, but may lack statistical strength in some cases. A prior constraint on EP values that weakens as TERM statistics become better defined appears to be an effective solution for combining the strengths of the two metrics.

**Table 2:** Prediction performance under different models.

Model^a^	*R*	*ρ*	RMSE	Accuracy
TERM-ΔΔ*G*	0.556 / 0.720	0.541 / 0.734	1.207	0.745
ConPot	0.479 / 0.669	0.422 / 0.643	1.274	0.689
TERM-ΔΔ*G*_*x*_	0.474 / 0.658	0.495 / 0.692	1.278	0.739
TERM-ΔΔ*G*_0_	0.309 / 0.523	0.310 / 0.537	1.381	0.687
TERM-ΔΔ*G*_1_	0.553 / 0.722	0.524 / 0.725	1.210	0.733
TERM-ΔΔ*G*_2_	0.564 / 0.729	0.541 / 0.736	1.199	0.747

The second number in columns *R* and *ρ* corresponds to removing 10% of data points as outliers. In all models needing λ, it was set to 1000.

^a^ Model tested. TERM-ΔΔ*G*: the full TERM-based model from Table 1 (λ = 1000).

ConPot: uses no TERM data and simply sets pEPs to be the contact potential and sEPs to be zero;

TERM-ΔΔ*G*_*x*_: sets the default values for all EPs to be zero, using TERM data as the only evidence for EP determination.

TERM-ΔΔ*G*_0_: considers only statistics from order-0 sub-TERMs (secondary structures);

TERM-ΔΔ*G*_1_: considers only statistics from order-0 and order-1 sub-TERMs;

TERM-ΔΔ*G*_2_: considers only statistics from order-0, order-1, and order-2 sub-TERMs. The latter is used as the standard model in the rest of the study.

### Contribution of higher-order tertiary structural motifs

In developing our model, we reasoned that statistics emergent from complex queries, those that simultaneously encompass multiple contacts of the mutated residue, would be important for correctly capturing the relevant structural environment. It is known that protein thermodynamics cannot be accurately modeled with independent pairwise additive inter-residue contributions [50, 51]. And although our framework does not attempt to model many-body interactions explicitly, it does require self and pair EPs to be optimized simultaneously in the context of all queried sub-TERMs. Thus, in match ensembles of complex sub-TERMs, multiple pEPs combine to evaluate amino-acid preferences at the mutated position (see Eq 3). In this way, parameters change concomitantly during optimization, which has the potential to capture couplings between contributions of multiple contacts, to some extent. Of course, this assumes that observations of relevant multi-contact sequence patterns are sufficiently abundant. To test whether statistics from complex sub-TERM ensembles really did benefit prediction, we considered models in which ensembles of some high-order sub-TERMs were removed.

When only zero- and first-order sub-TERMs were preserved, corresponding to one- and two-segment motifs only, prediction performance decreased relative to the full model (Table 2). Interestingly, however, a model that considered only zeroth-, first-, and second-order sub-TERM ensembles performed slightly better than the full model; see Table 2 and corresponding scatter plot in Fig 2. This suggests that the PDB is now large enough to allow capturing meaningful statistics for the coupling between two contacts, which are effectively three-body contributions. However, the benefit of mining for even higher-order correlations appears diminished. On the one hand, such couplings are expected to be small from physical principles (though not necessarily negligible). On the other hand, the representation of associated higher-order fragments is expected to be poorer. As a result, incorporation of data from such higher-order motifs does not appear to be beneficial at the moment, though it does not significantly degrade performance either. Perhaps unsurprisingly, zero-order sub-TERMs alone perform rather poorly (see Table 2, model TERM-ΔΔ*G*_0_), despite having the highest number of structural matches and best-resolved sequence statistics. This marked difference between TERM-ΔΔ*G*_0_ and either of the higher-order models demonstrates that tertiary-structure information encoded in TERMs contributes greatly to prediction accuracy.

**Fig 4:**
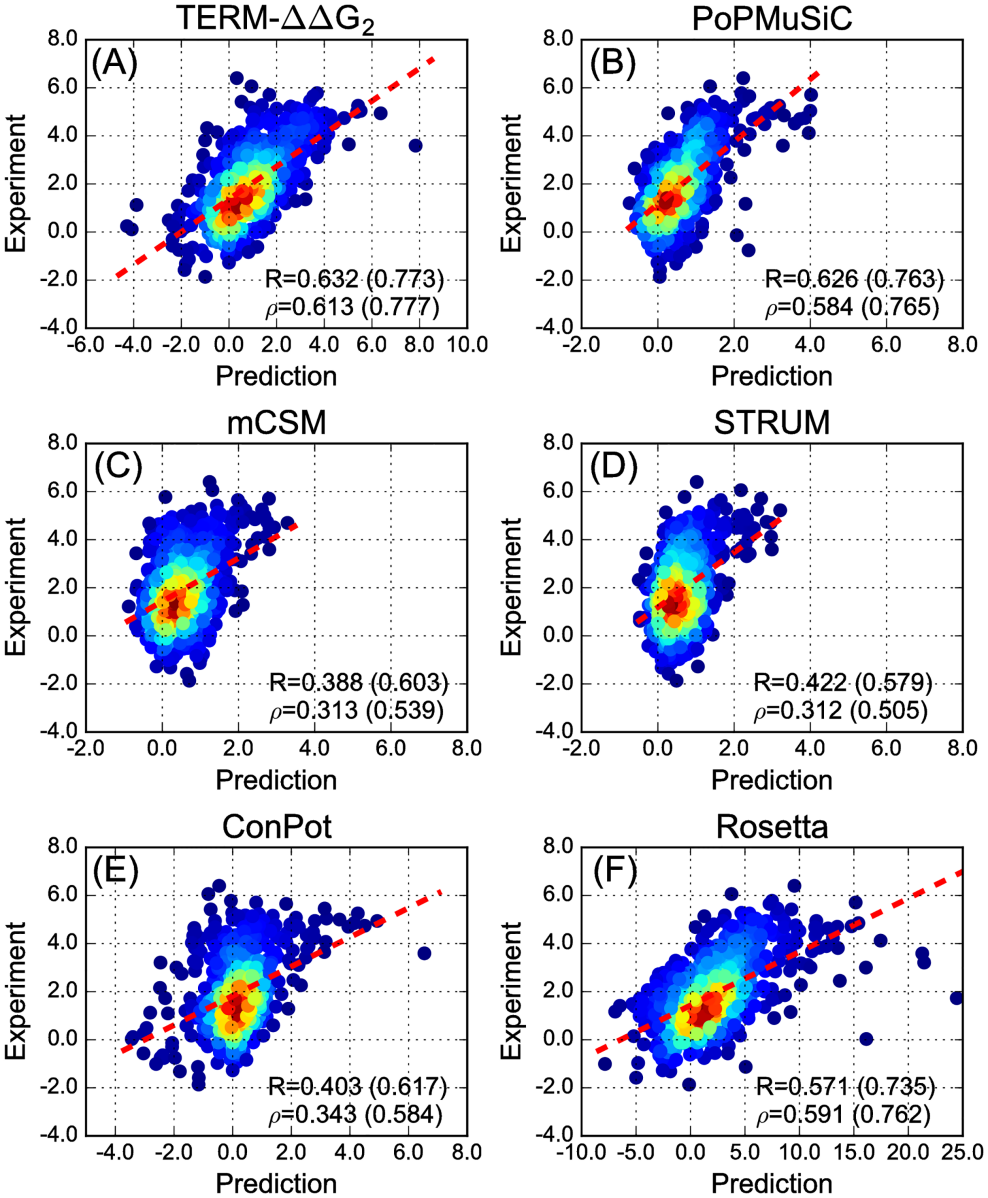
The performance of different methods on the S699 set. Data in each pannel are shown in the same manner as in Fig 2, with panel title indicating the prediction method used.

Inclusion of multi-contact ensembles appears to better model mutations in crowded positions, where considering the contribution of each contact independently may cause an overestimation of the total effect. A representative example of this is the mutation I8A in 1RIS (ribosomal protein S6 from *Thermus thermophilus*). This position is in a *β*-strand, surrounded by several other *β*-strands and an *α*-helix, with a total of nine contacting positions by our definition (Fig 3A). Considering only local and first-order sub-TERMs strongly over-predicts the effect of mutating Ile to Ala, by simply adding the (generally unfavorable) contributions emergent from the statistics of each individual contact. On the other hand, when second-order sub-TERMs are included (a total of 36 in this case), effectively forcing pair EPs to be consistent with one another, the contributions of individual contacts decrease, and the final prediction closely agrees with the experimental value (see Fig 3). This appears to be a general trend, with mutations at more crowded positions (i.e., those with more available second-order sub-TERMs) exhibiting higher improvement in ΔΔ*G*_*m*_ prediction and more reduction of pEP magnitudes upon the addition of order-two sub-TERMs, compared to their less crowded counterparts (Fig 4A in S1 Text).

Because of its comparable performance relative to the full model and the shorter running time, we chose TERM-ΔΔ*G*_2_ as the standard model for the rest of the study. Its performance metrics on the S2648 are *R* = 0.564, *ρ* = 0.541, RMSE = 1.199, Accuracy = 0.747 (Fig 2), and if 10% of outliers are discarded (by to the same procedure as in Dehouck *et al*. [37]), *R* and *ρ* increase to 0.729 and 0.736, respectively (Table 2).

### Comparison with existing methods

In developing their ΔΔ*G*_*m*_ prediction model, PoPMuSiC 2.0, Dehouck *et al*. used 2298 mutations from S2648 to learn (via neural networks) 27 scaling factors for combining 26 statistical energy contributions, setting the remaining 350 mutations aside for testing [37]. The same test set (S350) has been used as a standard benchmark by several other leading predictors, such as mCSM and STRUM [41, 42]. The performance of TERM-ΔΔ*G*_2_ on S350 (*R* = 0.62, *R* = 0.78 with 10% outlier exclusion) is comparable to those of PoPMuSiC, mCSM, and STRUM (Table 3), while other predictors tested by Dehouck *et al*. showed considerably lower performance [37].

**Table 3:** The performance of the TERM-based model relative to other published methods.

Method	*R* (S350 set)	*ρ* (S350 set)	*R* (S699 set)	*ρ* (S699 set)
TERM-ΔΔ*G*_2_	0.623 / 0.779	0.595 / 0.773	0.632 / 0.773	0.613 / 0.777
PoPMuSiC2.0 [37]	0.671 / 0.807	0.651 / 0.815	0.626 / 0.763	0.584 / 0.765
mCSM [41]	0.700 / 0.823	0.660 / 0.820	0.388 / 0.603	0.313 / 0.539
STRUM [42]	0.79 ^a^	N/D	0.422 / 0.579	0.312 / 0.505
Rosetta [52]	N/D	N/D	0.571 / 0.735	0.591 / 0.762
ConPot	0.567 / 0.716	0.481 / 0.663	0.403 / 0.617	0.343 / 0.584

Where listed, the second number in columns *R* and *ρ* corresponds to removing 10% of data points as outliers. N/D—not determined.

^a^ Taken directly from Quan *et al*. [42].

Importantly, our method does not involve training on experimental ΔΔ*G*_*m*_ data. Instead, it produces estimates based on the fundamental assumption that TERM sequence statistics are shaped by an underlying thermodynamic process. Thus, the extent to which the approach produces meaningful ΔΔ*G*_*m*_ values is a validation of this hypothesis. Further, because it requires no training on prior ΔΔ*G*_*m*_ data, the method is not subject to artifacts that can emerge in machine-learning models due to systematic biases in training/testing data. Predictors of ΔΔ*G*_*m*_ have generally been trained and tested on data from ProTherm [49]—a high-value resource for the field, which nevertheless has a biased representation of mutations. For example, 28% (744) of the mutations in S2648 are substitutions to Ala, as protein stabilities and functions are often probed with alanine-scanning experiments. Also, mutations from large to small side chains (i.e., those decreasing molecular weight by the equivalent of one -CH_2_- group or more) are dominant in S2648, making up 76% (2000) of all mutations. Finally, training biases can be introduced through the overlap between positions in the training and test sets. Our survey of S350 found that 71 out of 300 unique positions in this set also occurred among the 2298 mutations that remained after excluding S350 from S2648, each four times on average. Thus, the standard training and test sets used by many existing predictors are not truly independent. For all of these reasons, we sought to determine whether our TERM-based framework offers advantages over other predictors in scenarios where the mutations of interest are more diverse.

A truly stringent and unbiased test would be to predict the “thermodynamic landscape” of a protein—i.e., the ΔΔ*G*_*m*_ of all 19 possible mutations at all positions. Whereas direct experimental measurement of such landscapes is laborious, a growing number of recent studies have utilized deep sequencing technologies to systematically describe the effects of mutations on protein function, characterising “fitness landscape” instead [53]. Functional changes due to single mutations are not necessarily highly correlated with ΔΔ*G*_*m*_, as the wild-type protein is usually stable enough that stabilizing mutations may not offer further fitness gains, and mildly destabilizing mutations are not always deleterious enough to cause a detectable fitness loss. However, it has been shown that the stability effect of a mutation can be instead identified in the context of a marginally stable protein (i.e., with appropriate destabilizing mutations as the background), and sequence libraries of double mutations have been created for this purpose [54, 55].

**Fig 5:**
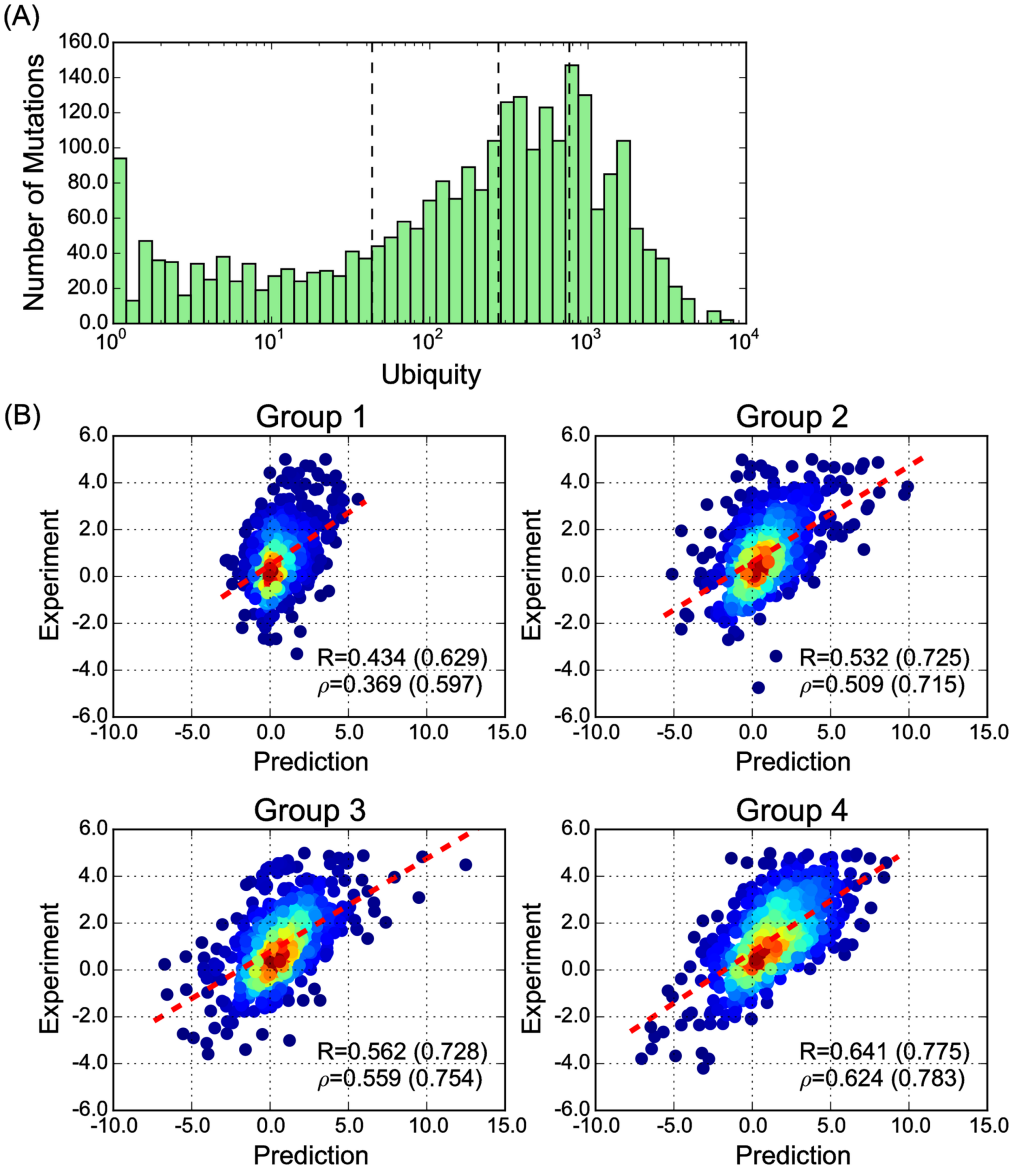
Abundance of structural information is critical to performance of prediction. (A) The distribution of ubiquity for mutations in the S2648 set. Quartile boundaries are labeled as dashed lines. (B) Performance of prediction on the four subgroups, from low ubiquity (group 1) to high ubiquity (group 4). The same representation is used here as in Fig 2.

Sun and co-workers have created a fitness landscape for all single and double mutations of the 56-residue protein GB1 (G immunoglobulin-binding domain B1; PDB entry 1PGA) by mRNA display and deep sequencing techniques, using the binding to IgG as the functional readout [55]. They also derived a method for estimating the ΔΔ*G*_*m*_ of a mutation based on its fitness effect in an appropriate genetic background of marginal stability ($\Delta \Delta G_{m}^{\text{fit}}$), showing it to closely correlate with thermodynamically measured ΔΔ*G*_*m*_ values (*R* > 0.9 for 82 mutations). Here, we took 699 high-confidence $\Delta \Delta G_{m}^{\text{fit}}$ estimates from the Wu *et al*. study as an unbiased benchmark dataset (S699) (see details in Materials and methods) to compare our TERM-based method with PoPMuSiC2.0, mCSM, and STRUM [55]. Our method performed the best in this test (*R* = 0.623, *ρ* = 0.601), closely followed by PoPMuSiC-2.0 (*R* = 0.626, *ρ* = 0.585). On the other hand, only weak correlations were observed between the $\Delta \Delta G_{m}^{\text{fit}}$ and predictions of mCSM and STRUM (Fig 4 and Table 3), despite both of these showing outstanding performance on S2648 and S350. As with previous test sets, the performance of ConPot (a simple residue-level contact potential) on S699 is worse than that of the TERM-based approach, but the difference is far greater here. This suggests that the impact of TERM statistics is increased in the case of an unbiased set of mutations (compare Tables 2 and 3). Further supporting this, the full TERM-ΔΔ*G* model, which considers all high-order sub-TERMs, marginally outperforms the standard TERM-ΔΔ*G*_2_ on this dataset (see Fig 5 in S1 Text).

The stark differences in performance of some methods on the two types of test sets may be due to training biases—explicit (i.e., in the fitting of model parameters) or implicit (i.e., in the choice of model type and relevant descriptors). For example, the loss of buried surface area and secondary-structure propensities, appropriately combined, may be reasonably sufficient for predicting the effects of mutations to Ala, but could be less adequate for predicting other substitution types (e.g., introduction/loss of a buried hydrogen bond). To distinguish more intricate differences between amino-acid energetic preferences, the explicit local structural environment, which can be captured using TERM match ensemble statistics, could be increasingly important.

An alternative means of predicting ΔΔ*G*_*m*_ values is to physically model the consequence of the mutation on the structure and compare the apparent change in stability of the folded state to that of the unfolded state. Though this is a difficult task, structure/physics-based methods for predicting ΔΔ*G*_*m*_ have been proposed [56–59]. Chief among the difficulties with such predictions is anticipating changes in backbone structure upon mutation, as well as balancing the contribution from different physical effects (e.g., burial of polar groups versus additional hydrogen-bond interactions). Recently, capabilities implemented within the Rosetta software suite [31] have enabled the exploration of structure-based models that do explicitly account for structural deformations, and also aim to capture the various energetic contributions with a library of both physical and empirical energy terms. In a recent study, Park *et al*. systematically optimized the energy function within Rosetta by adjusting its various parameters to improve performance on a range of structure modeling tasks, including single-mutation ΔΔ*G*_*m*_ prediction [52]. The resulting optimal energy function, used in conjunction with a new thorough backbone sampling protocol, showed a large improvement in ΔΔ*G*_*m*_ prediction over Rosetta’s previous standard energy function *talaris2014* [52]. We thus compared the performance of our TERM-based approach to that of this optimized Rosetta protocol as representing the state of the art in explicit structure-based modeling for ΔΔ*G*_*m*_ prediction. As shown in Fig 4F, the Rosetta-based approach performs better than some of the statistical methods or the simple ConPot model (*R* = 0.571, *ρ* = 0.591), but the TERM-ΔΔ*G*_2_ approach performs better still (predictions of TERM-ΔΔ*G*_2_ on S350 and S699 are provided as S1 Data).

### Prediction accuracy depends on the amount of structural data

The accuracy of our ΔΔ*G*_*m*_ prediction varies considerably for different mutations. Understanding the origin of this variation could enable estimation of prediction quality and may suggest avenues for further model improvements. We reasoned that the accuracy of TERM-based ΔΔ*G*_*m*_ estimation should be affected by the amount of available structural data matching the local environment of the mutated position. To quantify this, we introduced the metric *ubiquity* for a mutation from *u* to *v* at position *i*, which captures how well the sequence/structure context of the mutation is represented in sub-TERM matches:

$$\nu \left(u,v\right)=\max\left[\frac{O(J_{u}^{i})}{20}+\frac{1}{\left|C\right|}\sum_{j\in C}O\left(J_{u,\sigma_{0}^{j}}^{ij}\right),\frac{O(J_{v}^{i})}{20}+\frac{1}{\left|C\right|}\sum_{j\in C}O\left(J_{v,\sigma_{0}^{j}}^{ij}\right)\right]$$ (8)

Here, the two inner terms capture the effective average occurrence of the EPs involved in modeling either of the amino acids at the site. Function max represents the intuition that if one amino acid is well represented in TERM statistics while the other is poorly represented, then there is sufficient evidence to predict that the former should be preferred at the position. On the other hand, if both amino acids are poorly represented, then estimating an accurate difference would seem difficult. We divided the S2648 set into its four quartiles by ubiquity (distribution shown in Fig 5A) and analyzed performance separately in each quartile. As shown in Fig 5B, performance increased markedly and monotonically with ubiquity across these subsets, suggesting that sufficient relevant structural data are indeed critical for prediction accuracy.

**Fig 6:**
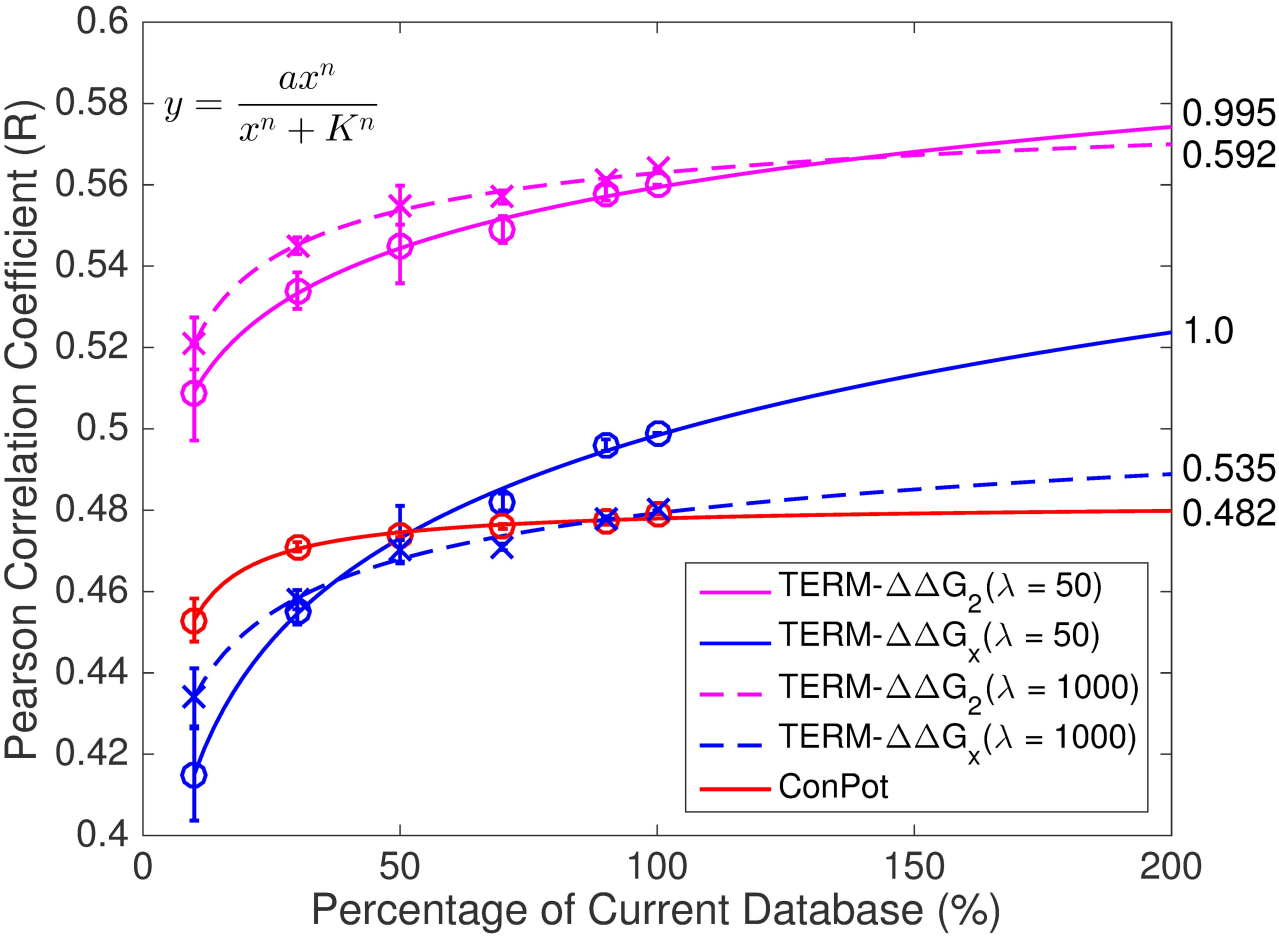
Prediction performance increases with the size of the structural database. The model represented by each curve is indicated in the legend. For each level of subsampling, three samples were generated, with error bars showing the standard deviations among the three trials for each experiment. The functional form used in fitting is shown in the upper-left corner. The numbers on the right side of each curve indicate the corresponding best-fit plateau values (i.e., parameter *a*).

Given the above, we should expect the performance of our predictions to increase as the amount of data in the PDB grows. To test this idea, we randomly subsampled the non-redundant PDB subset used throughout this study to 10%, 30%, 50%, 70% and 90% of its size, and re-ran the same prediction procedure as described above each time. To truly simulate a smaller PDB, we also re-derived the contact potential (i.e., default values for pEPs) for each subsampled subset. Once again, the performance on S2648 increased monotonically with growing database size (Fig 6). Note, that similar trends were observed with different λ values (50 and 1000), so the increase is not merely an artifact under a specific strength of regularization.

One can ask whether the increasing performance is mainly due to better ensemble statistics or a better contact potential (i.e., better default pEPs). To discriminate between these two possibilities, we also considered the ConPot and TERM-ΔΔ*G*_*x*_ models (contact potential only and zero prior on EP values, respectively, as defined previously) for each structural subset. This revealed that better ensemble statistics were the primary driving force for performance improvements with larger structural subsets, whereas even 30% of structural data produced a contact potential that performed roughly as well as that from the entire database (Fig 6).

It is tempting to use the relationship between database size and prediction accuracy to extrapolate what performance one might expect as the PDB continues to grow. We observed that this relationship is well described by the Hill equation, and thus used this functional form to perform extrapolations (see Materials and methods). As shown in Fig 6, extrapolated performances suggest that TERM-based statistics will become more useful as structural data continue to accumulate. Models that involve weak regularization (λ = 50) are predicted to eventually outperform all those with strong regularization (λ = 1000), suggesting that it will become increasingly advantageous to use sequence statistics from explicitly-defined structural environments (i.e., TERMs and sub-TERMs) rather than “mean-field” statistics of simple geometric descriptors. Interestingly, the value of the best-fit plateau parameter from the Hill equation is near unity for models with weak regularization (i.e., perfect performance in the limit of infinite data), but not those with strong regularization. Fig 6 also shows that the performance of ConPot has already saturated, suggesting that contact potentials will not improve with more data. And whereas using ConPot values as priors helps for now (e.g., compare TERM-ΔΔ*G*_2_, λ = 50 and TERM-ΔΔ*G*_*x*_, λ = 50), their impact is predicted to diminish with database size. Whether these extrapolations are borne out in the future remains to be seen. Nevertheless, it is interesting that current trends point towards TERM-based mining of sequence-structure becoming increasingly useful.

## Discussion

The primary goal of this study is to test the hypothesis that PDB-derived sequence statistics of tertiary structural motifs reflect fundamental sequence-structure relationships. We examine this idea by considering the problem of predicting ΔΔ*G*_*m*_ based solely on sequence preferences of sub-structures surrounding the mutated position. Instead of describing sequence propensities associated with elementary structural descriptors like distances or angles, as in traditional statistical potentials, we extract sequence statistics directly from explicit, complex structural units—TERMs and sub-TERMs. This has the potential benefit of inherently representing correlations between elementary features, taking advantage of the modular nature of protein structure space.

The significant correlation between TERM-derived and experimentally-determined ΔΔ*G*_*m*_ clearly supports our central hypothesis, demonstrating that TERMs can indeed be seen as encoding rules of sequence-structure compatibility. Further, the performance of TERM-based ΔΔ*G*_*m*_ itself is noteworthy, as it is on par with or exceeds that of alternative approaches. The current state-of-the-art in this problem is represented by statistical or machine-learning methods, which combine a variety of structural descriptors and train on existing thermodynamic mutational data. In contrast, the TERM-based approach does not involve training and simply rests on the assumption of the relevance and correctness of TERM sequence statistics. If TERM statistics were too noisy, strongly affected by database biases, or simply captured irrelevant effects, the prediction would work poorly. In this sense, the fact that the method is competitive with alternatives tuned for the task clearly validates PDB-derived tertiary structural statistics as a novel quantitative metric, which although not devoid of biases, should nevertheless be useful in practice with broad applicability.

Methods for ΔΔ*G*_*m*_ prediction have generally relied on the ProTherm database for training/testing. An invaluable large compendium of protein thermodynamic data, the database nevertheless represents a biased set of mutations, strongly skewed towards those more frequently attempted in standard biophysical analyses (e.g., mutations to Ala). This appears to have affected the generality of some previously-developed statistical methods (see Table 3). For example, the mCSM and STRUM approaches [41, 42] perform far better than any other tested method on the S350 subset from ProTherm (an “industry standard” training set), reaching an impressive *R* of 0.7 and 0.79, respectively, but far underperform relative to other models on the unbiased S699 set, with an *R* of 0.39 and 0.42, respectively. In contrast, the performance of the TERM-based approach is nearly identical on both sets. Interestingly, the decrease in performance from S350 to S699 is considerably smaller for PoPMuSiC2.0 [37]—another approach based on machine learning. We think this is because PoPMuSiC2.0, unlike the other two statistical methods tested here (mCSM and STRUM), uses statistical potential terms (e.g., torsion-angle and inter-residue distance potentials) among its descriptors. Because these are derived from a representative subset of the PDB, they are generalizable beyond any specific ΔΔ*G*_*m*_ training set.

Predicting ΔΔ*G*_*m*_ for mutations that increase side-chain bulk is known to be challenging [56], especially for physics-based models. It is difficult to differentiate between cases where the backbone rearranges to accommodate the mutation without affecting (or even improving) stability, relative to those where the additional bulk is incompatible with the folded ensemble and destabilizes the structure. Although explicit sampling of backbone degrees of freedom, e.g., as implemented in the Rosetta modeling suite [31], allows for the potential to recognize backbone relaxations upon mutation, it is unclear *a priori* which of the many possible sampling approaches and energy functions would work best for a given system [59]. Recently, Park *et al*. developed a new energy function in Rosetta, which showed better performance in a wide range of structure modeling applications, including ΔΔ*G*_*m*_ prediction [52]. The performance of this method on the unbiased S699 set is respectable (considering the difficulty of predicting ΔΔ*G*_*m*_ with explicit structural sampling), but does suggest that explicit modeling of backbone rearrangement remains a significant challenge (see Table 3). By representing the local environment around the mutation with structural ensembles, the TERM-based approach has the potential to implicitly account for backbone relaxation. On the other hand, the ensembles may also be too fuzzy and forgiving to recognize truly destabilizing perturbations. The fact that TERM-based predictions perform well in both stabilizing and destabilizing regimes (see Fig 4A) indicates that the former effect dominates over the latter, and that TERM match ensembles may be an effective means of recognizing the plasticity of the backbone.

Our analysis also revealed a clear association between prediction accuracy and the amount of available relevant structural data (see Fig 5). This enables our method to produce a metric of confidence (ubiquity, see Eq 8 and Fig 5A), along with the prediction itself, which is of clear utility in a practical setting. For example, in a bioengineering setting, if the goal is to improve the stability of a protein and two mutations have a similar predicted stabilizing ΔΔ*G*_*m*_, knowing which result is likely to be more accurate can greatly aid in prioritizing experiments. The association between accuracy and the amount of available data also means that as the structural database continues to grow, we can expect our estimator to perform increasingly better. This effect can be clearly seen through subsampling the current structural database, enabling an extrapolation into the future to suggest how the performance *may* improve as the database continues to grow (Fig 6).

Beyond providing more TERM instances, the growing structural database will increasingly enable the consideration of more complex motifs. Our results indicate that tertiary motifs (i.e., sub-TERMs with two or more disjoint segments) are key to capturing correct sequence-structure relationships and achieving high ΔΔ*G*_*m*_ prediction performance. Inclusion of more complex multi-segment motifs generally improves performance, but appears to plateau after order-2 sub-TERMs (i.e., those with three segments). This is likely not because more complex TERMs are not important, but rather because their statistics become limiting. A larger structural database would enable more such motifs to be considered, such that future accuracy improvements may be larger than can be anticipated with a simple extrapolation. Further, some target proteins (or mutated positions) may be composed of more canonical structural patters, which would warrant inclusion of more complex motifs even now. Indeed, the average ubiquity of the S699 set is higher than that in S2648, and the “complete” model TERM-ΔΔ*G* (which considers all possible sub-TERMs, provided they have sufficient statistics) exhibits marginally better prediction performance on this set than the standard TERM-ΔΔ*G*_2_ model (see Fig 5 in S1 Text).

We have previously shown that protein structure space is highly modular at the level of TERMs, with as few as 652 motifs sufficient to describe over half of the structural universe at sub-Angstrom resolution [20]. Together with the fundings in the present study, tying TERM sequence statistics to thermodynamic determinants of structure, this suggests that the apparent modularity of structure space may be due to the limited number of stable local structural environments that are available in the context of naturally occurring amino acids.

## Conclusion

Stability is a filter in the evolution of proteins, so sequence statistics associated with any structural motif, large or small, must at least be consistent with thermodynamic principles. On the other hand, many other considerations bias the choice of sequence through evolution (e.g., function). Further, the portion of the protein structural universe that is available to us is biased by many factors, including limitations of structure determination. We set out to investigate whether sequence propensities emergent from structural matches to local tertiary motifs, TERMs, could be seen as encoding fundamental sequence-structure relationships or whether the various biases and limited statistics would make these ineffective in practice. The task of predicting ΔΔ*G*_*m*_ is ideal for resolving this question. This is a challenging problem, as illustrated by the limited performance of even the best methods, so serendipitously good correlations on large datasets are extremely unlikely. The strong performance of TERM statistics in predicting ΔΔ*G*_*m*_ implicate thermodynamic principles as a major driving force behind TERM formation and their sequence preferences. In our view, this finding has both fundamental implications towards our understanding of the modularity in the protein structural universe, and applied implications for statistics-based modeling of protein energetics. Statistical potentials have made tremendous impact towards protein modeling, and our results suggest that a new kind of statistics-based model of protein sequence-structure relationships may be possible—one based on localized sequence models describing the preferences of individual complex structural motifs comprising a protein in question.

## Materials and methods

### Contact degree

The metric of contact degree between two positions, introduced in our earlier studies [20, 46], was computed as follows:

$$c\left(i,j\right)=\frac{\sum_{a=1}^{20}\sum_{b=1}^{20}\sum_{r_{i}\in R_{i}\left(a\right)}\sum_{r_{j}\in R_{j}\left(b\right)}I_{ij}\left(r_{i},r_{j}\right)Pr\left(a\right)Pr\left(b\right)p\left(r_{i}\right)p\left(r_{j}\right)}{\sum_{a=1}^{20}\sum_{b=1}^{20}\sum_{r_{i}\in R_{i}\left(a\right)}\sum_{r_{j}\in R_{j}\left(b\right)}Pr\left(a\right)Pr\left(b\right)p\left(r_{i}\right)p\left(r_{j}\right)}$$ (9)

where *i* and *j* are the two positions, *R*_*i*_(*a*) is a set of side-chain rotamers of amino acid *a* at position *i* (after discarding rotamers that clash with the backbone), *I*_*ij*_(*r*_*i*_, *r*_*j*_) is a binary variable indicating whether the two rotamers *r*_*i*_ and *r*_*j*_ would likely strongly influence each other’s presence (have non-hydrogen atom pairs within 3 Å), *Pr*(*a*) is the frequency of amino acid *a* in the structural database (see Table 1 in S1 Text), and *p*(*r*_*i*_) is the probability of rotamer *r*_*i*_. Rotamers and their probabilities were taken from the backbone dependent library developed by Dunbrack and coworkers [60]. Positions *i* and *j* were said to be poised to interact (i.e., their amino-acid choices were expected to be strongly inter-dependent) if *c*(*i*, *j*) exceeded 0.02.

### Structural ensembles and redundancy removal

Matching ensembles for each motif were obtained by using MASTER [47] to search structure fragments in a curated list of 16,967 non-redundant PDB chains from X-ray structures with resolution of 2.5 *Å* or better [46], in which the sequence identity between any two chains was under 30%, and peptide chains (those with fewer than 30 residues) were excluded. The search was performed by full-backbone RMSD, with the cutoff chosen automatically, as reported previously [20], based on the complexity of the query motif (see S1 Text).

To ensure that our results reflected purely structure-based principles and not homology relationships, TERM match ensembles were post-processed to remove matches from proteins that appeared to share even remote homology, locally or over the entire chain, to the protein under study (i.e., the protein whose mutations were being estimated). Specifically, the chain sequence of the studied protein was searched against the PDB using the BLASTpgp program in BLAST2.26 [61], and proteins giving rise to any alignments with an E-value below 1.0 were defined as homologous and marked for removal from the match ensemble. This effectively removed homology to the studied protein, but did not deal with the redundancy of the structural database. Although we were already dealing with a redundancy-pruned subset of the PDB (see above), redundancy on the local level was still possible. To address this, we extracted the local sequence around the seed residue of every match in the ensemble of a given TERM, taking 15 positions on each side of the seed position or a 31-residue window. We then used the program Usearch [62] to efficiently cluster these 31-mer sequences at 40% sequence identity, keeping only a single match from within each resulting cluster (the one with the lowest RMSD to the query). The clustering was performed with the following command:

usearch8.0 –cluster_fast in_file –id 0.4 –centroids out_file –fulldp –query_cov 1 –target_cov 1 –maxgaps 0

where in_file included one-letter amino-acid sequences of all the 31-aa windows (one per line) and out_file contained the clustering results.

### Contact potentials

All contacting residue pairs from our non-redundant PDB subset (i.e., pairs with contact degrees above 0.02) were collected and further sub-divided into 5 bins by contact degree: [0.02, 0.05), [0.05, 0.1), [0.1, 0.2), [0.2, 0.5), [0.5, 1]. Within each bin, we calculated the statistical potential fo interaction between amino acids *a* and *b* as follows:

$${\hat{J}}_{ab}=-\ln\frac{N_{obs}\left(a,b\right)}{N_{exp}\left(a,b\right)}$$ (10)

where *N*_*obs*_(*a*, *b*) and *N*_*exp*_(*a*, *b*) are the observed and expected number of occurrences of the amino-acid pair in the bin, respectively. Whereas *N*_*obs*_(*a*, *b*) is simply given y the data, appropriately evaluating *N*_*exp*_(*a*, *b*) is critical for a statistical potential. A naive approach is simply multiplying the background frequencies of *a* and *b* by the total number of contacts in each bin. However, it has been shown that this approach erroneously evaluated the magnitudes of inter-residue forces [50]. For example, due to the closely packed nature of protein interiors, many pairs of hydrophobic residues in proximity are merely “by-standers” of “real” attractive interactions (much fewer in number). Therefore, the burial state of residue pairs needs to be considered to properly estimate the background expectations.

To this end, we introduced a sequence-independent metric of residue burial, called *freedom*, which ranges from 0 to 1 and describes the fraction of rotamers of all amino acids at a given position that can be accommodated (without clashes) in the context of the backbone alone (see S1 Text for details). This metric effectively captures the amount of free space available around a given residue that is relevant for placing side-chains. We computed freedom for all residues in our structural database, dividing them into 70 equally-spaced freedom bins to compute amino-acid freedom propensities. Given this, the expected number of amino-acid pair *a*, *b* in a given contact-degree bin was computed as:

$$N_{exp}\left(a,b\right)=\frac{1}{1+I\left[a=b\right]}\cdot \sum_{i=1}^{N_{obs}}\left[P\left(a|F_{k_{i}}\right)\;P\left(b|F_{l_{i}}\right)+P\left(b|F_{k_{i}}\right)\;P\left(a|F_{l_{i}}\right)\right]$$ (11)

where $N_{obs}=\sum_{a=1}^{20}\sum_{b=1}^{20}N_{obs}\left(a,b\right)$ is the total number of contacts found in the bin, *k*_*i*_ ad *l*_*i*_ are the two positions forming the *i*-th contact, respectively, *P*(*a*|*F*_*k*_*i*__) is the probability of observing *a* at position *k*_*i*_ given the freedom bin corresponding to the freedom value of position *k*_*i*_ (*F*_*k*_*i*__). *I* [*a* = *b*] is a binary indicator variable, which evaluates to unity if *a* and *b* are the same amino acid and to zero otherwise. This is needed because the terms under the sum in Eq 11, which compute the expectation of observing the amino-acid pair *a*, *b* at a given pair of positions, account for both occurrences of *a*, *b* and *b*, *a*, which overcounts by a factor of two if *a* and *b* are the same.

### Extraction of energy parameters

With our automated RMSD cutoff function (see S1 Text), the predominant majority of MASTER searches (92.5% of those for S2648 with model TERM-ΔΔ*G*, see Fig 1 in S1 Text) returned fewer than 20,000 matches. Thus, to reduce computational cost, for larger match ensembles, only the top 20,000 matches were kept in the stage of EP extraction. The objective function in Eq 7 was minimized in MATLAB, using the function *fminsearch* in the Optimization Toolbox, which implements a Nelder-Mead simplex search algorithm. To speed up the optimization, EPs were subdivided into 1 + |*C*| groups, each containing either 20 sEPs or 400 pEPs for one of the |*C*| contacts, with optimization performed sequentially for each EP group. During optimization for one group, the EPs in other groups were held fixed. One cycle of optimization involved visiting each group once, and this was repeated until either 1) the improvement of objective function between consecutive cycles was smaller than 10^−5^ or 2) the *l*^2^-norms of EP value differences between adjacent cycles were smaller than 0.01 for all groups.

### Performance metrics

Pearson (*R*) and Spearman (*ρ*) correlation coefficients were calculated with standard formulas. The RMSE was computed by first performing a linear regression using predicted and experimental values as the independent and dependent variables, respectively, and then computing the root mean squared deviation between the experimental values and the optimally-transformed predicted ones.

In each test, we report *R*, *ρ*, and RMSE for the entire dataset as well as after removal of most outlying 10% of the points. Outliers were removed iteratively in a greedy fashion, by finding the point that most improves the corresponding metric at each iteration and removing it.

### Calculating ΔΔ*G*_fit_ from the GB1 fitness landscape

Sun and co-workers used an mRNA display technique in combination with deep sequencing to characterize the relative binding efficiency of all single and and nearly all double mutants of IgG-binding domain of protein G (GB1) to IgG-Fc [55]. To this end, the frequency of each variant *i* was measured before and after one generation of affinity enrichment, defining the fitness score of the variant, *W*_*i*_, relative to wild type as:

$$W_{i}=\frac{N_{i,\text{input}}}{N_{\text{i,}\;\text{selected}}}\cdot \frac{N_{wt,\text{selected}}}{N_{wt,\text{input}}}$$ (12)

where *N*_*i*,input_ and *N*_i, selected_ are the raw sequence counts of variant *i* before and after enrichment, respectively, and *N*_*wt*,selected_ and *N*_*wt*,input_ are the same for the wild type, respectively. The authors showed that these data enable one to quantitatively estimate the $\Delta \Delta G_{m}^{\text{fit}}$ of a given mutant B via the formula [45, 55]:

$$\Delta \Delta G_{m}^{\text{fit}}\left(B\right)=-\ln\frac{W_{\text{B}}-W_{\text{AB}}}{W_{\text{AB}}}+\ln\frac{1-W_{\text{A}}}{W_{\text{A}}}$$ (13)

where A was a reference mutation and *W*_AB_ the fitness of the *A*, *B* double mutant, showing their estimates to be highly correlated (*R* > 0.9 for 82 mutations) with biophysically-measured stability changes. As discussed in detail by the authors [45], the optimal reference mutation is that, which exhausts the stability margin of the protein, such that any further change in stability could be accurately estimated based on fitness changes. For the specific choice of GB1, Sun and co-workers found five such reference mutations (Y3A, Y3C, L5N, L5S, F30N) and we used all five in our analysis. Taking the raw counts from the original study [55], we computed the above estimate for all substitutions (for which there were data) using all five reference mutations, and further applied several quality filters. An estimate was discarded if 1) *W*_B_ ≤ *W*_AB_, as this violated an implicit assumption behind deriving Eq 13, 2) *N*_AB, input_ < 90, to remove noise from low sequencing counts (this exclude ∼ 10% of double mutants with the lowest sequencing counts), or 3) *W*_B_ < 0.24, since Olson *et. al* observed that $\Delta \Delta G_{m}^{\text{fit}}\left(B\right)$ were most accurate when substitution *B* had sufficient fitness to give the dynamic range for estimating (*W*_B_ − *W*_AB_)/*W*_AB_. After applying all of the filters, an estimate based on at least one background mutation survived for 699 mutations, with the final $\Delta \Delta G_{m}^{\text{fit}}$ for each taken to be the average over all available background mutations.

### Performance as a function of database size

To subsample our structural database, we randomly chose 10%, 30%, 50%, 70%, or 90% of the chains comprising the initial non-redundant database. Each level of subsampling was independently repeated 3 times. To test a given subsample, the same procedures as elsewhere were employed for structural searching, data collection and pruning (i.e., for redundancy and homology), and extraction of energy parameters, but using a reduced database. For models involving the contact potential as a prior, the potential was re-calculated from the reduced set of contacts corresponding to subsampled database.

The relationship between database size and and the Pearson correlation coefficient (see Fig 6) was fit to the Hill equation, *a* ⋅ *x*^*n*^/(*x*^*n*^ + *K*^*n*^), where *x* is the fraction of subsampling, *a* is the maximal attainable performance value, *n* is the Hill coefficient, and *K* is the database size at which half the maximal attainable performance is expected. Fitting was performed using the function *fit* in MATLAB. Adjustable parameters (i.e., *a*, *n*, and *K*) were constrained to be non-negative and *a* was constrained not to exceed 1 (maximal possible *R* value) by adjusting the fitting function to be min(|*a*|, 1) ⋅ *x*^|*n*|^/(*x*^|*n*|^ + |*K*|^|*n*|^) and interpreting optimal parameters accordingly. A total of 100 fittings were attempted for each curve, starting with random parameters, and the values giving the lowest sum squared error were reported. Repetition of this procedure produced in very similar values, suggesting that the fitting was robust.

### Code availability

Code and running instruction for performing TERM-based prediction of ΔΔ*G*_*m*_ can be found at http://grigoryanlab.org/terms/.

## Supporting information

S1 Text Supplementary Methods, Supplementary Figures 1-5, Supplementary Table 1. (PDF)

S1 Data A spreadsheet containing predictions of model TERM-ΔΔ*G*_2_ the S350 and the S699 mutation sets. (XLSX)
