# Supplementary material for: Sequence statistics of tertiary structural motifs reflect protein stability
Source: PLoS One. 2017 May 26;12(5):e0178272. doi: 10.1371/journal.pone.0178272 (PMC5446159; doi:10.1371/journal.pone.0178272)
Supplement: S1 Text — (PDF) [file pone.0178272.s001.pdf]

Supplementary Information for Zheng *et al.*, "Sequence statistics of tertiary structural motifs reflect protein stability"

May 18, 2017

Supplementary Methods

RMSD Cutoff

The RMSD threshold c for close matches of a motif is defined as the following:

$$\begin{aligned} d &= N\left(1 - \frac{2}{N(N-1)} \sum_k \sum_{i=1}^{n_k} \sum_{j=i+1}^{n_k} e^{(i-j)/L}\right) \\ c &= \sigma_{max}\sqrt{d/N} \end{aligned} \tag{1}$$

where d is the effective number of degrees of freedom for the TERM, n_k is the length of the k-th segment of the motif, N is the total length of the motif (i.e. $N = \sum_k n_k$) , L is correlation length—a parameter describing the extent of positional correlation between two residue in the same polypeptide chain, and σ_{max} is the RMSD threshold parameter. See MacKenzie *et al.* for a detailed discussion and derivation of this universal RMSD cutoff [1]. In this work, L and σ_{max} were chosen to be 18 and 1.0 Å, respectively.

Metric of Residue Burial

We used the metric *freedom* to quantify the burial state of a residue. The freedom of position i, F_i is calculated as:

$$p_c(r_i) = \sum_{j \neq i} \sum_{b=1}^{20} \sum_{r_j \in R_j(b)} I_{ij}(r_i, r_j) Pr(b) P(r_j) \tag{2}$$

$$V_{i,\tau} = \frac{\sum_{a=1}^{20} \sum_{r_i \in R_i(a)} I(p_c(r_i) < \tau)}{\sum_{a=1}^{20} |R_i(a)|} \tag{3}$$

$$F_i = \sqrt{\frac{V_{i,0.5}^2 + V_{i,2}^2}{2}} \tag{4}$$

Definitions of $r_i, R_i(a), Pr(a), P(r_i)$ and $I_{ij}(r_i, r_j)$ are the same as those in Equation 9 from the main text. $p_c(r_i)$ is the "collision probability mass" of rotamer r_i—i.e., how likely it is to clash with rotamers at other positions. Higher $p_c(r_i)$ values indicate that r_i is expected to be generally crowded out by other side-chains in its environment. $V_{i,\tau}$ then quantifies the total weight of rotamers at position i that are not overly crowded (i.e., have collision probabilities below τ). Finally we combined $V_{i,\tau}$ with two threshold τ, 0.5 and 2, into the final freedom metric F_i.

Effects of Order-2 Sub-TERMs

Improvement upon switching from model TERM-$\Delta\Delta G_1$ to model TERM-$\Delta\Delta G_2$, for a particular mutation k, was calculated as $|P_{k,1} - E_k| - |P_{k,2} - E_k|$, where E_k was the experimental $\Delta\Delta G_m$ value for the mutation, while $P_{k,1}$ and $P_{k,2}$ were those predicted with TERM-$\Delta\Delta G_1$ and TERM-$\Delta\Delta G_2$, respectively. The fraction of weakened pair interactions (Fig 4B) was evaluated as the fraction of pairwise contributions to predicted $\Delta\Delta G_m$ (i.e., differences between pEPs corresponding to wild-type and mutated amino acids) whose magnitude decreased by at least 10% when switching from TERM-$\Delta\Delta G_1$ to TERM-$\Delta\Delta G_2$; weak pairwise contributions (i.e., those with magnitudes below 0.5 in the initial model) and those that changed sign upon model switching were discarded. The results of these analyses are shown in Fig 4.

Rosetta $\Delta\Delta G_m$ prediction

Rosetta $\Delta\Delta G_m$ prediction was performed using the version of the suite downloaded from the RosettaCommons `github.com` repository on Nov. 30, 2016, by running the "cartesian_ddG" protocol according to the instructions described by Park et al. [2].

Supplementary References

[1] Mackenzie CO, Zhou J, Grigoryan G. Tertiary alphabet for the observable protein structural universe. Proc Natl Acad Sci U S A. 2016 Nov;.

[2] Park H, Bradley P, Greisen Jr P, Liu Y, Mulligan VK, Kim DE, et al. Simultaneous Optimization of Biomolecular Energy Functions on Features from Small Molecules and Macromolecules. Journal of Chemical Theory and Computation. 2016;.

Supplementary Figures

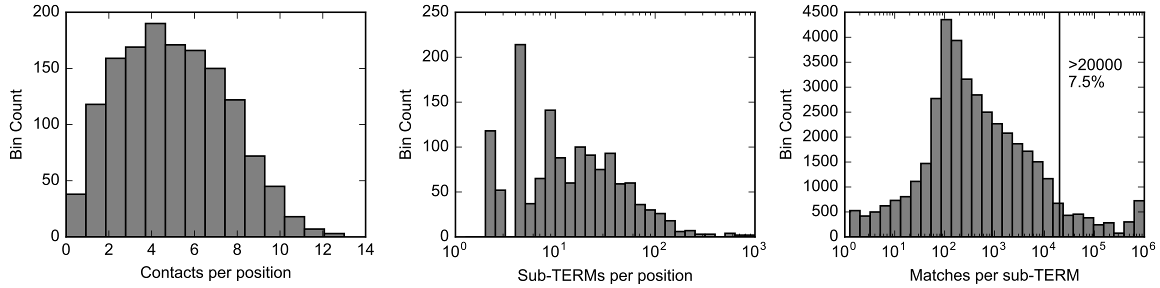

Fig 1. Statistics of the S2648 mutation set. Shown left to right are histograms of number of positions labeled as contacting the mutated position by our contact degree definition, total number of sub-TERMs considered for each mutation in our framework, and number of structural matches per sub-TERM, respectively.

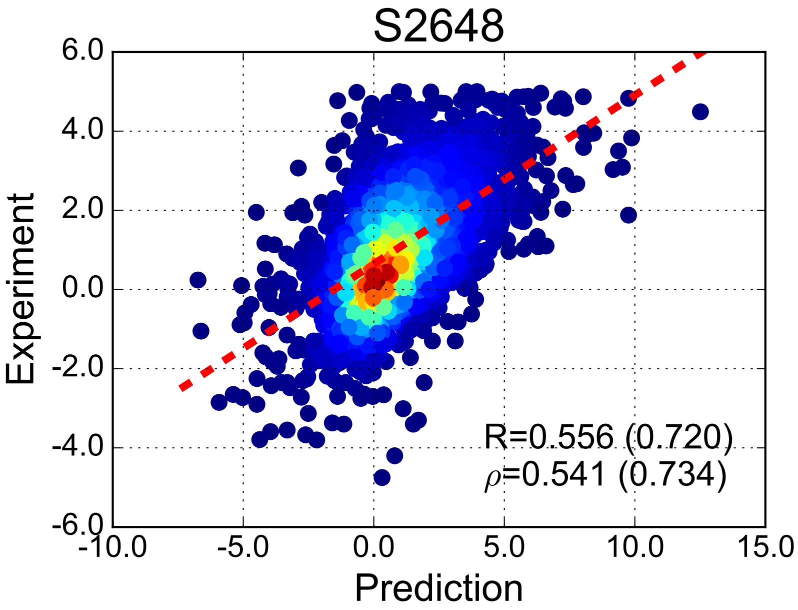

Fig 2. The performance of TERM-$\Delta\Delta G$ on S2648. Predicted and measured $\Delta\Delta G_m$ values are plotted on the X- and Y-axes, respectively. Color represents point cloud density. The least-squares regression line is shown with dashes.

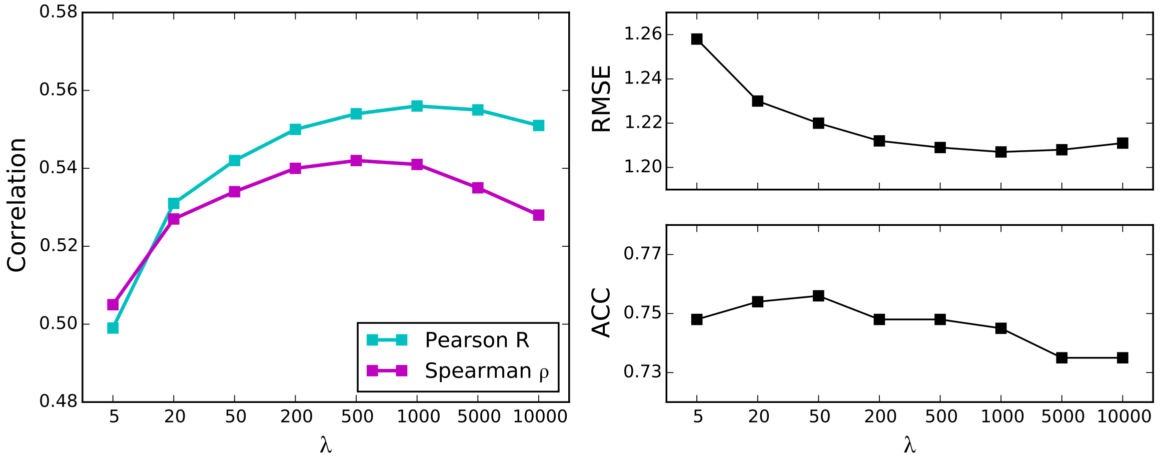

Fig 3. Performance of prediction under different strengths of regularization. Pearson and Spearman correlation coefficients, RMSE, and ACC are reported for λ value of 5, 20, 50, 200, 500, 1000, 5000, and 10000.

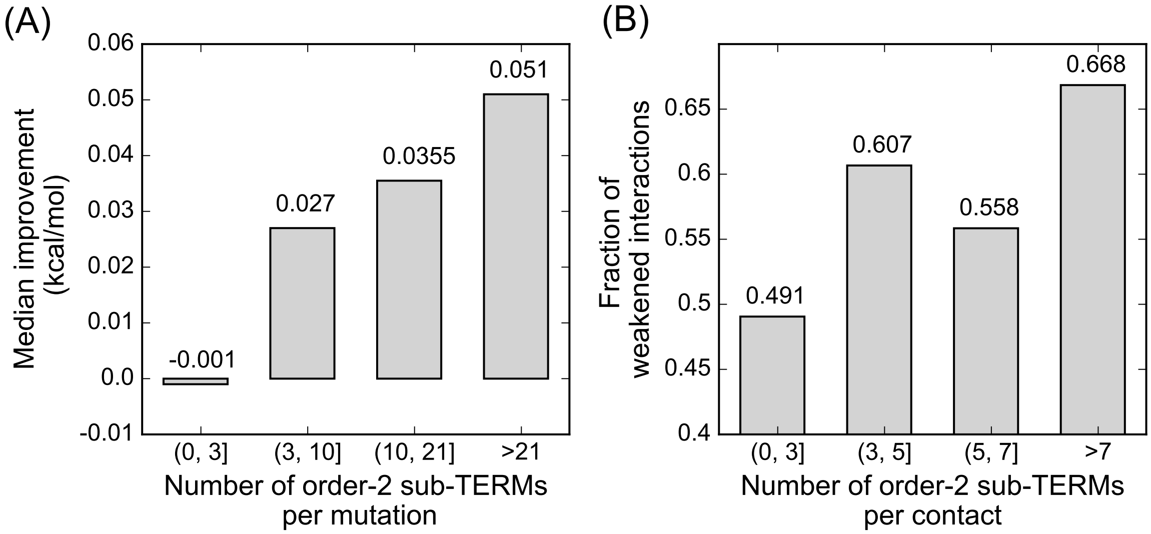

Fig 4. Including order-2 sub-TERMs (i.e., switching from model TERM-$\Delta\Delta G_1$ to model TERM-$\Delta\Delta G_2$) improved the prediction for multi-contact positions (see Supplementary Texts). (A) Predicted $\Delta\Delta G_m$ values for mutations with more order-2 sub-TERMs (X-axis) tend to move towards corresponding experimental values (Y-axis) more significantly upon switching the model. (B) The magnitudes of pair contributions to $\Delta\Delta G_m$ predictions decrease more frequently for mutations with more order-2 sub-TERMs (x-axis) upon switching the model.

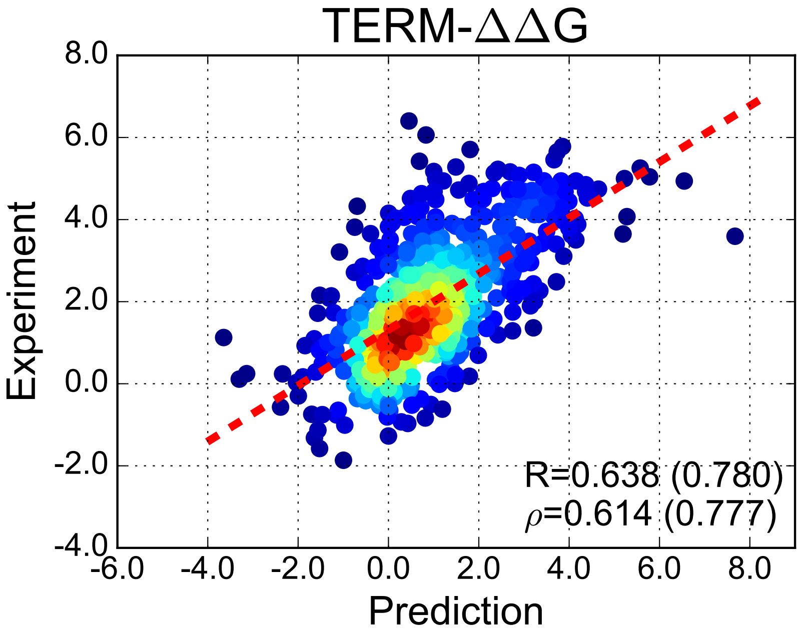

Fig 5. The performance of TERM-$\Delta\Delta G$ on S699. Data are shown in the same manner as in Fig 2.

Supplementary Tables

Table 1. Background frequency of amino acids.

Amino acid	Frequency	Amino acid	Frequency
ALA (A)	0.0795	CYS (C)	0.0133
ASP (D)	0.0589	GLU (E)	0.0684
PHE (F)	0.0410	GLY (G)	0.0692
HIS (H)	0.0234	ILE (I)	0.0582
LYS (K)	0.0584	LEU (L)	0.0955
MET (M)	0.0217	ASN (N)	0.0433
PRO (P)	0.0451	GLN (Q)	0.0382
ARG (R)	0.0520	SER (S)	0.0602
THR (T)	0.0542	VAL (V)	0.0699
TRP (W)	0.0139	TYR (Y)	0.0357
